# Advances in Electrospun Nanofiber Membranes for Dermatological Applications: A Review

**DOI:** 10.3390/molecules29174271

**Published:** 2024-09-09

**Authors:** Yuanyuan Han, Hewei Wei, Qiteng Ding, Chuanbo Ding, Shuai Zhang

**Affiliations:** 1 College of Traditional Chinese Medicine, Jilin Agriculture Science and Technology College, Jilin 132101, China; 18343249985@163.com; 2 College of Chinese Medicinal Materials, Jilin Agricultural University, Changchun 130118, China; www03211@126.com (H.W.); ding152778@163.com (Q.D.)

**Keywords:** carbohydrate polymer, nanofiber membrane, skin diseases, electrostatic spinning

## Abstract

In recent years, a wide variety of high-performance and versatile nanofiber membranes have been successfully created using different electrospinning methods. As vehicles for medication, they have been receiving more attention because of their exceptional antibacterial characteristics and ability to heal wounds, resulting in improved drug delivery and release. This quality makes them an appealing choice for treating various skin conditions like wounds, fungal infections, skin discoloration disorders, dermatitis, and skin cancer. This article offers comprehensive information on the electrospinning procedure, the categorization of nanofiber membranes, and their use in dermatology. Additionally, it delves into successful case studies, showcasing the utilization of nanofiber membranes in the field of skin diseases to promote their substantial advancement.

## 1. Introduction

The primary organ of the body is the skin, which serves as the initial barrier against foreign microbes, providing physiological and mechanical support to underlying cells and organs while preventing the entry of foreign substances. Any injury or infection can compromise this protective layer, resulting in various skin conditions and autoimmune diseases like atopic dermatitis, skin pigmentation disorders, acne, and skin cancer. Tissue regeneration is the key to repairing damaged skin, involving four distinct stages: hemostasis, inflammation, proliferation, and remodeling [1]. Throughout the healing process, the extensive surface area of the skin can be utilized to aid in the delivery of nanoparticle systems and nanofiber membranes for the targeted treatment of skin diseases through drug loading.

Carbohydrate polymers, such as chitosan or alginate, display robust adhesive properties. This feature enhances the retention of nanodelivery systems on the skin, extends drug release duration, and permits the creation of nanoparticles or hydrogels based on carbohydrate polymers with regulated drug release capabilities [2]. The encapsulation of anticancer medications in nanostructures enables controlled drug release, leading to improved efficacy, reduced side effects, and enhanced therapeutic outcomes. Carbohydrate polymer-based nanodelivery systems can deliver multiple therapeutic drugs simultaneously, allowing for combination therapy. Capitalizing on the intrinsic properties of carbohydrates, nanofiber membranes offer numerous possibilities for skin applications.

Researchers have extensively investigated how to achieve the precise delivery of active compounds to the skin by utilizing various nanodelivery systems, such as metal nanoparticles, liposomes, solid lipid nanoparticles, nanoemulsions, and carbon nanomaterials [3]. The application of nanotechnology extends to the treatment of various medical conditions such as cardiovascular disease, infectious diseases, gastrointestinal disorders, neurodegenerative diseases, pain management, and wound healing. The fabrication of nanofibers involves a range of manufacturing techniques, including electrospinning, phase separation, physical fabrication, and chemical synthesis.

By utilizing electrospinning technology, the nanofiber membrane exhibits remarkable antibacterial properties and efficacy in skin penetration and wound healing processes [4]. It shows significant inhibitory effects on Staphylococcus aureus (99.86% antibacterial efficacy) and *Escherichia coli* (99.7% antibacterial efficacy) without harming human skin fibroblasts [5,6,7]. In addition, the filtration efficiency can be improved by optimizing the filtration medium to achieve better antibacterial performance [8,9]. By making adjustments to the nanofiber membrane properties at the molecular level, researchers have successfully developed a new transdermal drug delivery system with excellent biocompatibility and improved drug-loading capacities [10]. The drug-laden nanofiber membrane, fabricated through electrospinning, presents an opportunity for treating skin conditions by facilitating the development of a robust vascular endothelium, ensuring sustained barrier functionality for prolonged drug release [11]. With high porosity, interconnectivity, and flexibility, nanofibers are well-suited for targeted therapy against various skin conditions, including cancer, bacterial infections, inflammation, and wounds.

## 2. Preparation Method of Nanofiber Membrane

### 2.1. Electrospinning Principle

Nanofibers are primarily composed of various polymer types, with their size or diameter depending on the type of polymer, manufacturing technique, and design specifications. Electrospinning stands out as the most widely employed method for nanofiber membrane preparation, involving key components like a syringe pump, high-voltage power source, and ground collector. Operationally, the polymer solution (or melt) is dispensed from a syringe under a constant speed via a pump, while an electric field is applied by the high voltage source [12]. The stable electrospinning jet comprises distinct zones: the base, jet, fan, and collection regions. The geometry of the jet is influenced by the surface tension of the liquid. Electrostatic charging occurs at the inception of the jet, forming a structure known as the Taylor cone [13]. During the electrospinning process, fibers are placed onto a collector that is grounded. Once the fibers are deposited, the electrical charge on the optical fiber dissipates rapidly through the collector. Despite the low conductivity of the solution, a significant residual charge remains on the collected fiber surface [14]. Electrospinning technology, which is based on polymer preparation, is categorized into solution electrospinning and melt electrospinning [15]. The electrospun fibers create an interconnected porous network, allowing for high gene loading and sustained release over a prolonged period.

### 2.2. Electrospinning Classification

Initially used for producing nanofibers for textiles [16] and filtration [17], electrospinning now has crucial applications in various fields. It is utilized in food packaging to improve food preservation by leveraging its antibacterial and antioxidant properties [18,19,20]. Additionally, it enhances the encapsulation efficiency of active substances, immobilizes enzymes, coats edible food, and enables intelligent packaging. It is also vital for thermal management composites using phase change materials [21,22]. Numerous polymers, such as poly-L-lactic acid (PLA), polycaprolactone (PCL), polyurethane (PU), polyvinylidene fluoride (PVDF), and polyhydroxy butyrate hydroxy valerate (PHBV), have been electrospun into nanofibrous structures.

As the original method for producing nanofibers, electrospinning has advanced from a single-fluid blending process [23] to include coaxial [24,25], triaxial [26], side-by-side [27], and other multi-fluid processes [28]. These methods yield core–shell [29,30,31] and merged nanostructures [32]. In parallel, electrospinning is integrated with various chemical and physical techniques to expand the creation of novel functional polymer materials [33]. The following are several electrostatic spinning processes, advantages and categories for treating skin diseases (Table 1).

#### 2.2.1. Coaxial Electrostatic Spinning

The concept of coaxial electrostatic spinning uses two distinct propulsion mechanisms to control the spinning solution in both the central and outer layers, respectively. A specialized coaxial needle tip facilitates the convergence of two layers of solution. Typically, the outer layer advances twice as quickly as the inner layer to ensure the proper wrapping of the core solution by the shell solution under the influence of an electric field. This process ultimately yields nanofibers with a core–shell configuration as the solvent evaporates [41].

**Figure 1:**
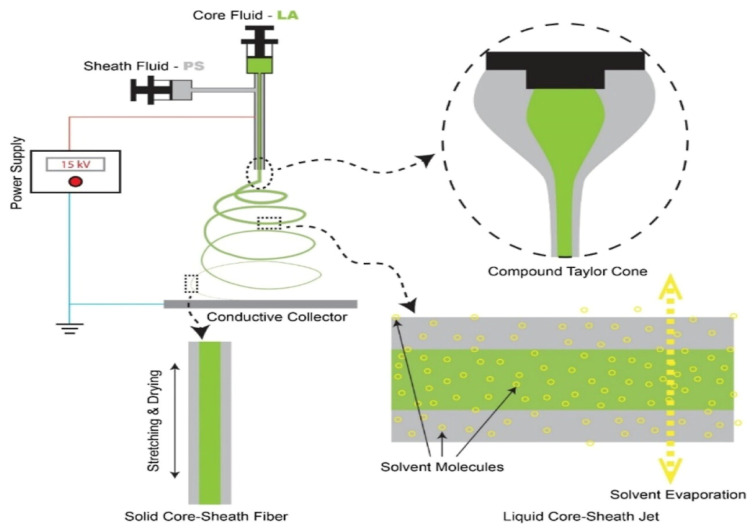
Schematic illustrations showing the coaxial electrospinning process for encapsulating LA into PS by forming LAPS core–sheath nanofibers [44], with permission from ACS publications.

In contrast to single-nozzle electrospinning, coaxial electrospinning must also consider additional factors, such as the feeding rate of the core–shell solution and solution compatibility. Current research has investigated the production of PVDF nanofiber membranes with optimal morphology and high electrical properties using electrospinning [42]. The successful implementation of coaxial electrostatic spinning relies on the compatibility of the core and shell solutions to establish a stable Taylor cone at the spinneret’s tip [43]. This compatibility has been demonstrated in experimental settings. For example, a study conducted on the efficient and eco-friendly encapsulation of natural phase-change materials within nanofibers through coaxial electrospinning introduced a method of encapsulating natural phase-change materials (LA) in polystyrene (PS) nanofibers [44] (Figure 1).

Furthermore, electrospun nanofibers aligned in a coaxial structure have shown improved cell proliferation and precise control over release kinetics for drug delivery applications. Xu and colleagues [45] utilized coaxial electrospinning technology to fabricate core–shell fibers composed of silk fibroin/polylactic acid–caprolactone–polyethylene oxide for the delivery of growth factors such as fibroblast growth factor 2 and connective tissue growth factor. An in vitro release test showed that the initial burst release amount was 37.6 ± 1.8% in the first 8 h, and the cumulative release amount reached 81.7 ± 1.8% on the seventh day. On the 7th day, the release curve showed that the release rate was less than 1% every day, and on the 14th day, the cumulative release amount of the shell reached 91.6 ± 1.8%.

The unique configuration of coaxial electrospun nanofibers enables the production of materials beyond traditional electrospinning, allowing for tailored core and shell materials to meet various application needs. This setup also allows for adjustments to process instruments and electrospinning conditions [46].

#### 2.2.2. Side-by-Side Electrostatic Spinning

**Figure 2:**
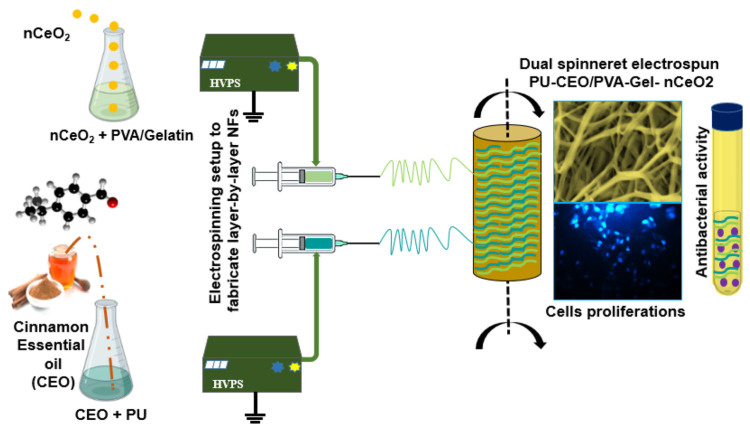
Schematic representation of the setup for the dual spinneret electrospun scaffolds PU-CEO/PVA-Gel-nCeO_2_ [35], with permission from ACS publications.

The exploration of side-by-side electrospinning began relatively recently when Gupta and Wilkes pioneered the preparation of PVC/PVDF and PVC/Estane bicomponent fibers using parallel spinnerets in 2003 [47]. This method overcame the challenge of blending and electrospinning two solutions to obtain bicomponent fibers. In contrast to the conventional core–sheath structure, the two chambers in side-by-side electrospinning are distinct and interact independently with the surrounding environment. Variation in the properties of nanofibers can be achieved through the design of the spinneret structure and adjustments to electrospinning parameters [48]. Zheng et al. investigated the impact of dual drug loading in electrospun Janus nanofibers on the controlled release of tamoxifen (TAM) and elucidated the underlying mechanisms. An analysis of the drug release profile revealed the significant influence of different polymer components, two-compartment structures, and shapes on the in vitro release characteristics of TAM, highlighting their importance in designing functional nanomaterials [49]. In addition, Hussein et al. researched and developed a dual spinneret electrospinning technique that was applied to fabricate a series of polyurethane (PU) and polyvinyl alcohol–gelatin (PVA/Gel) nanofibrous scaffolds [35] (Figure 2). The obtained results indicate that the proposed PU/PVA-Gel NFs represent promising platforms with CEO and nCeO_2_ for effectively managing diabetic wounds.

Additionally, researchers devised a dual-layer mixed drug delivery system for the sublingual administration of therapeutic peptide desmopressin [50]. A hybrid system of mucoadhesive electrospun chitosan–polyethylene oxide nanofibers loaded with peptides (average diameter of 183 ± 20 nm) and a saliva-repellent backing film was developed to enable one-way release to the mucosa. In vitro release studies demonstrated the unidirectional release of desmopressin from the nanofiber system, with approximately 80% of the loaded peptide released within 45 min. The nanofiber–membrane hybrid system effectively protected peptides from being rinsed away. This was demonstrated in a porcine sublingual mucosa flow retention model, where 90% of the loaded desmopressin remained on the mucosal surface after exposure to simulated saliva flow for 15 min.

#### 2.2.3. Triaxial Electrostatic Spinning

Advancements in nanotechnology have prompted researchers to explore more advanced electrospinning techniques beyond traditional structures [51]. Triaxial electrospinning, as demonstrated by Naveen et al., involves loading different substances into distinct layers of the nanofibers to achieve specific properties [52]. In their study, rhodamine B (RhB) and bovine serum albumin (BSA) were encapsulated in the outer and middle layers, respectively, using PLGA as the sheath solution, gelatin as the intermediate layer, and PCL as the core layer. This triaxial electrospinning approach produced nanofibers with superior mechanical strength and dual drug release capabilities.

A three-layer triaxial electrospinning system consists of a high-voltage power supply, spinneret, heat collector, and fluid-driven pump [53,54]. The triaxial electrospinning process utilizes three concentric needles in the spinneret to deliver external, intermediate, and core liquids driven by separate pumps, allowing for the production of three-layer nanofibers with precise control over drug release properties. The operational mechanism of this procedure mirrors that of various other electrohydrodynamic atomization techniques (like coaxial electrostatic spinning) [55]. Electrostatic spinning technology in three axes has been diversified. The central polymer is enveloped by two dissimilar polymer layers, and the central polymer is encased by two identical polymer layers. Additionally, there is a central polymer enclosed by an outer layer and a void region.

**Figure 3:**
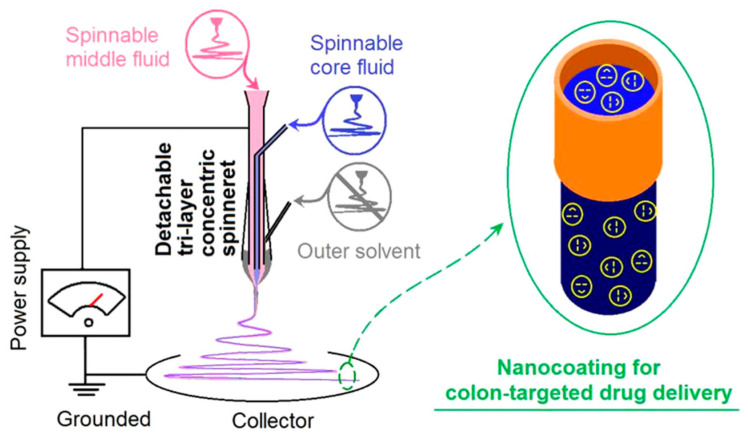
A schematic of the homemade triaxial electrospinning system [56], with permission from ACS publications.

Subsequently, Ding et al. [56] utilized the pH-sensitive polymer Eudragit S100 (ES100) as a matrix, loaded with the model drug aspirin, and fabricated core–shell nanofibers via enhanced triaxial electrospinning technology (Figure 3). In comparison to traditional single-fluid electrospun nanofibers, the in vitro drug release shows a sustained and extended aspirin release, preventing cytotoxicity from abrupt drug release. Following this, Zhao and colleagues adopted the group’s technological approach and employed the fabricated nanofiber membrane imbued with functional particles to explore antibiotic elimination in water [57]. Wang et al. illustrated that a drug reservoir was integrated into core–shell nanofibers through enhanced triaxial electrospinning technology. Utilizing cellulose acetate (CA) as the base polymer and acyclovir (ACY) as the model drug, an extended release period was observed in vitro. The core–shell configuration and non-uniform dispersion of the drug showcase an exceptional structure–performance correlation [26].

#### 2.2.4. Multi-Nozzle Electrostatic Spinning

Multi-nozzle electrospinning represents a strategy to mitigate the core deficiencies of conventional electrospinning [58]. The configuration of a multi-nozzle array can regulate the distribution of the electric field. Ensuring that the nozzles remain separate at their tips without merging requires a minimum spacing between the nozzles and the appropriate gap between adjacent nozzles. Adjusting the distance between the internal nozzles may reduce the repulsive force between them, while bringing them closer together can help concentrate the fibers. The spacing and arrangement of the nozzles in a particular area have a significant impact on the overall production throughput of fibers [59,60,61].

A multi-nozzle electrospinning system is designed to create large nanofibers for increased efficiency and coverage. Reports suggest that multi-needle electrostatic spinning has been used to create a skin–core architecture. The process of manufacturing nanofiber filaments includes two stages: spinning and stretching. The addition of an extra electrode during the spinning of nanofiber filaments helps to decrease interference from the electrostatic field between the needles, resulting in the reduced beam deflection and enhanced stability of the Taylor cone and beam. Electrospinning equipment equipped with multiple or fewer nozzles has the capability to produce streams of electrospun nanofibers [59].

By utilizing multi-nozzle electrospinning machinery, diverse polymer compositions can be electrospun to produce a blend of nanofiber mats with consistent thickness and ample dispersibility. It is also plausible to yield a mixed nanofiber mat with multiple polymers using this technique. A bottom-up nozzle array electrospinning system has been developed by CNR-ISMAC, featuring a 50 cm broad metal collector with 31 to 62 nozzles. These arrangements enable the formation of overlapping deposition regions to ensure uniform fiber deposition on the collector [62].

#### 2.2.5. Emulsion Electrostatic Spinning

**Figure 4:**
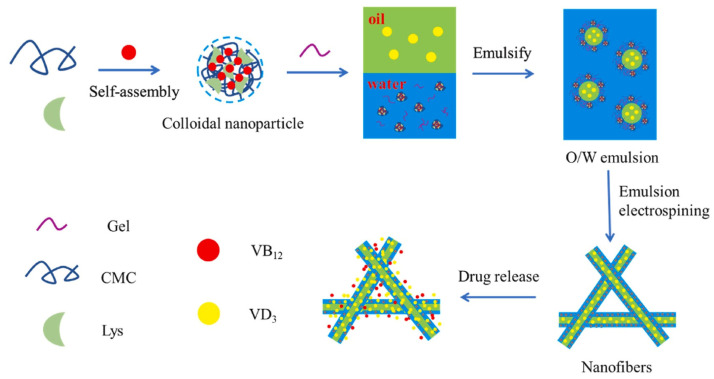
Dual drug-loaded core–shell nanofibers membranes via emulsion electrospinning and their controllable sustained release property [65], with permission from Elsevier.

Emulsion electrospinning and coaxial electrospinning are innovative techniques used in the production of core–shell nanofibers. The outer shell of these nanofibers can effectively encapsulate and prevent the release of active ingredients located in the inner core [63]. In contrast with coaxial electrospinning, emulsion electrospinning offers the advantages of simple processing and control [64]. Generally, emulsions come in two forms: oil in water (O/W) and water in oil (W/O) [65] (Figure 4). Under specific conditions, an oil-in-water emulsion was created using polylactic acid–glycolic acid polymer (PLGA) with hexafluoroisopropanol as the oil phase, polyvinyl alcohol as the water phase, and aloe (AV) along with recombinant human epidermal growth factor (rhEGF) as components. This emulsion exhibited prolonged release properties, enhancing fibroblast regeneration and wound healing [66].

In trials involving the production of nano-macromolecules through emulsion electrospinning, metformin hydrochloride (MH) or metoprolol tartrate (MPT) were blended with polycaprolactone (PCL) and poly (3-hydroxybutyrate-3-hydroxyvalerate) fibers. It was found that PCL exhibited superior drug transportation capabilities compared to PHBV [67]. Basar et al. [68] utilized electrostatic spinning to convert a stable oil-in-water (O/W) emulsion into a nanofiber membrane comprised of polycaprolactone–gelatin, cross-linked with glutaraldehyde. The polycaprolactone–gelatin composite exhibited sustained drug-release capabilities up to a duration of 4 days, surpassing a solitary polycaprolactone nanofiber membrane. 

Furthermore, core–sheath structured nanofibers were fabricated through an oil-in-water emulsion involving polyvinyl alcohol (as the water phase) and plant bactericide (as the oil phase) [69]. The release profile of plant fungicides from the core of the nanofibers over 21 days indicated sustained release within 14 days. The core–sheath structured nanofibers comprising plant fungicides–polyvinyl alcohol displayed a remarkable 99.9% antibacterial efficacy against Staphylococcus aureus and Escherichia coli, underscoring the potent antibacterial properties of the encapsulated plant fungicides.

#### 2.2.6. Bubble Electrospinning

A groundbreaking technique known as bubble electrospinning has recently been introduced into the intricate realm of electrospinning methodologies. This innovative approach utilizes electricity to disrupt the surface tension of bubbles, which are formed on the polymer solution surface with the flow of air. The size and shape of the bubbles have an impact on surface tension [70]. Bubble dynamics have been a subject of interest for a long time [71], and polymer bubbles are frequently used in bubble electrospinning to create nanofibers [72]. Bubble electrospinning, originally developed for producing smooth fibers [73], is regarded as the most effective technique for generating functional nanofibers. These nanofibers have diameters that vary from several nanometers to hundreds of nanometers, and are created under high pressure. This method is considered superior to other nanotechnologies like chemical vapor deposition and needle-like electrospinning [74,75,76,77,78].

An innovative technology for bubble electrospinning implements a cone-shaped gas nozzle paired with an enhanced bubble electrospinning (MBE) technique using a copper solution reservoir, enabling the effective fabrication of SF nanofibers [79]. Moreover, a new method has been introduced for creating side-by-side dimer nanofibers using bubble electrospinning [80]. These nanofibers are composed of various hydrophilic and hydrophobic regions arranged in parallel along their axes. Nanofibers were successfully produced through electrospinning, incorporating a hydrophilic component such as polyvinyl alcohol (PVA) along with a hydrophobic component like poly (ε-caprolactone) (PCL) or nylon 6. This led to the creation of nanofibers with both hydrophilic and hydrophobic segments. This cutting-edge technique yields nanofibers with a minimal diameter of 101 nm in PVA/Nylon6 dimer nanofibers.

**Figure 5:**
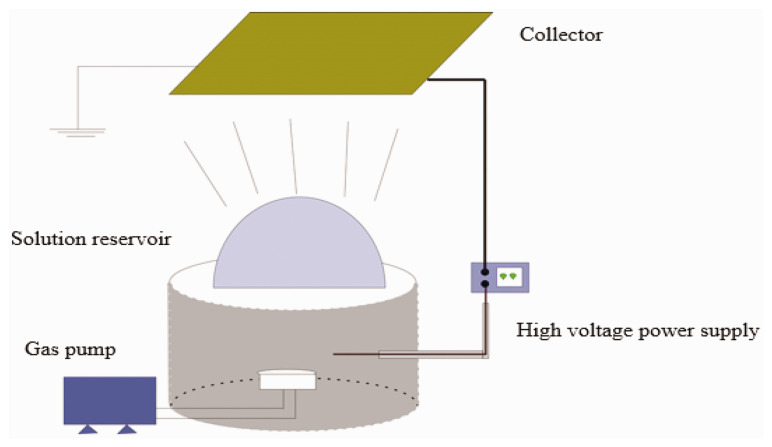
Device for critical bubble electrospinning [85], with permission from Sage Journals.

Bubble electrospinning technology has the potential for large-scale nanofiber production [81,82,83,84] and has found widespread application in the electrostatic spinning of PVA, PVP, PAN, and other polymers. For instance, a fabricated composite nanofiber membrane produced via the critical bubble electrospinning of polyacrylonitrile demonstrates capabilities for high-temperature adsorption and separation [85] (Figure 5).

## 3. Classification and Application of Nanofiber Membrane

Nanofibers encompass a wide array of materials, including polymers, ceramics, small molecules, and their blends. Apart from generating nanofibers with a smooth surface, electrospinning can also yield nanofibers with secondary structures such as pores, voids, core–shell configurations, and more. The arrangement of molecules or nanoparticles can modify both the surface and interior of nanofibers, whether carried out simultaneously during electrospinning or post formation. Nanofibers offer versatility in application, enabling the creation of various types of membrane structures. Specifically, this analysis focuses on PCL, PLA, PVP, and PLGA nanofiber membranes.

Nanofibers find extensive utility in different scenarios, including their integration onto fabric surfaces to establish stable gas films, yielding biphobic interface fabrics that are waterproof, oil-proof, and resistant to fouling. Furthermore, nanofibers are instrumental in the purification and filtration of chemical and pharmaceutical substances. Below, we delve into the drug-carrying capabilities of nanofiber membranes and their diverse application (Table 2).

## 4. Treatment of Dermatosis with Nanofiber Membrane

The evolving landscape of medical technology emphasizes the importance of administering multiple drugs simultaneously, with specific control over the rate and pattern of drug release. The realm of nanofiber membranes is at the forefront of skin wound treatment, advancing efforts to address the complexities of managing various skin ailments. Through electrospinning technology, nanofiber morphology transforms, allowing nanostructures to enable targeted drug delivery to the skin and minimize adverse effects.

### 4.1. Mycosis

Invasive mycosis presents a notable health concern for critically ill patients and individuals with weakened immune systems. The rise of fungal co-infections or super-infections has become a recent cause for alarm. Europe is currently facing high rates of candidiasis and aspergillosis, whereas India is experiencing a greater number of mucormycosis cases. Epidemiological evidence suggests a rising incidence of these three diseases. Additionally, certain fungal species may possess intrinsic resistance to azoles and other antifungal medications [105].

**Figure 6:**
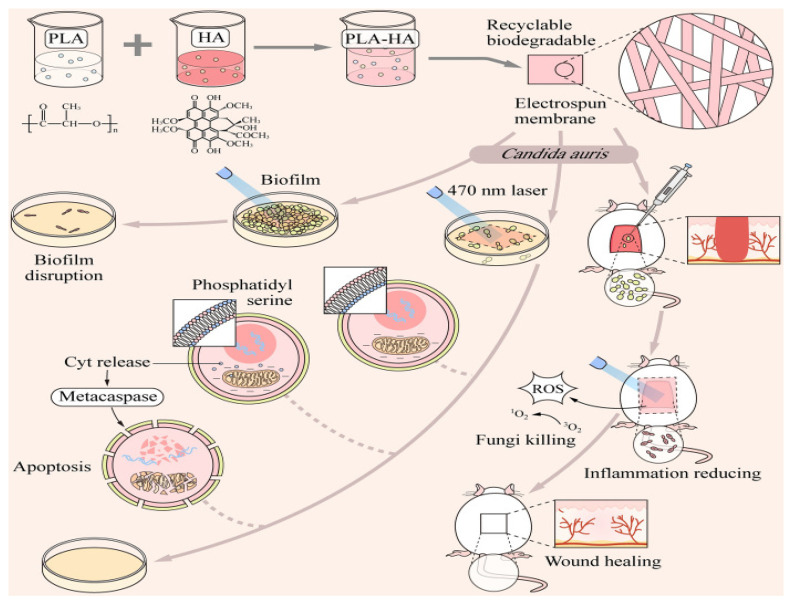
Schematic diagram of the synthetic route of PLA-HA membrane and its antifungal application. Class C phytoplankton and biofilm in oris and the healing of corresponding infected wounds [106], with permission from PLOS PATHOGENS.

Photodynamic therapy using antibacterial agents (aPDT) holds significant potential in clinical settings for the treatment of fungal infections. A biodegradable and reusable PLA-HA nanofiber membrane with light-responsive antifungal properties was successfully created via electrospinning. This unique nanofiber membrane, made of polylactic acid–oligothyroxine (PLA-HA), was developed and tested for its effectiveness in aPDT against oral fungal infections to explore the cellular mechanisms of cell death induced by PLA-HA-aPDT [106]. The synthesis pathway of the PLA-HA membrane and a visual representation of its antifungal application are depicted in Figure 6 [106]. Electrospun nano-membranes are particularly advantageous as antifungal drug delivery materials due to their improved air permeability, increased porosity and surface area, and greater capacity for loading non-toxic photosensitizers (PS), making them an ideal choice for defense against external microbial invasion [107,108]. By incorporating chitosan and a second-generation PS, an electrospun nanofiber membrane was developed to enable localized PDT sterilization upon exposure to visible light [109]. Polylactic acid (PLA), a synthetic polymer, is commonly used in fiber and film production as well as biomedical engineering due to its ability to be spun, induced crystallinity under tension, excellent biocompatibility, and biodegradability [110,111]. 

In order to fight against dermatophytes, Qasem Asgari et al. prepared electrospun core–shell nanofibers loaded with amphotericin B as a new dressing to treat superficial mycoses and cutaneous leishmaniasis [112]. Adel Al Fatease et al. prepared bio-curcumin (CMN) nanoemulsions (CMN-NEs) for transdermal drug delivery for the treatment of fungal diseases. The nanoemulsions (NEs) prepared using a self-nanoemulsification method possessed high skin permeability, while the CMN-NE gels enhanced the release of CMN from the gel matrix in mycobacterial patients [113]. Simone Jacobus Berlitz et al. aimed to develop a lipid-based nanocarrier containing CQ for dermal applications, and developed a nanopreparation containing CQ (LBN-CQ) and evaluated its antifungal activity using sonication to achieve therapeutic effects [114].

By carefully selecting the biopolymer characteristics used in producing electrospun fibers, the stability and bioavailability of bioactive components can be enhanced, facilitating their controlled release [115]. Effectively produced through the electrospinning process, electrospun fibers encapsulated various biopolymers and demonstrated potential as a post-harvest antifungal solution for commercially relevant fungi. The antifungal properties of spore germination and mycelium growth were examined through morphology, heat, chemistry, and in vitro analyses, revealing a significant enhancement in the antifungal efficacy of the nanofiber membrane generated by electrospinning fibers [116]. 

### 4.2. Skin Infection

Dermatomycosis affects exposed skin, the scalp, nails, and toenails, with oral and genital mucosa also being susceptible to fungal infections. The primary pathogen responsible for skin fungal infections is dermatophyte, specifically dermatophyte anthropophagus [117]. While antibiotics are commonly effective against bacterial infections, their overuse and misuse have led to the rise of drug-resistant bacteria. Exploring non-antibiotic alternative therapies and applying bioactive glass nanoparticles or nanocellulose to skin tissue may offer better results [118].

**Figure 7:**
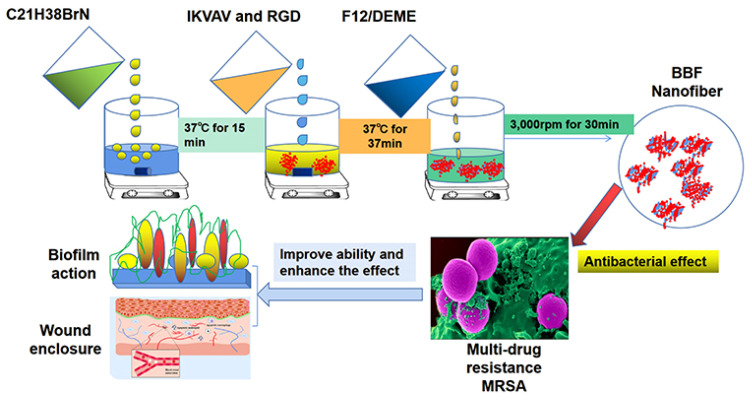
Schematic diagram of research on new bioactive antibacterial nanofibers and benzalkonium bromide [119], with permission from Dove Medical Press Limited.

Nanofibers have the capability to enhance cell adhesion, proliferation, and differentiation, with novel nanofiber wound dressings significantly accelerating wound healing. By employing a new self-assembly technique, Benzalkonium bromide (BZK) was effectively embedded into a novel nanofiber, as illustrated in Figure 7. The antibacterial efficacy, inhibition of biofilm formation, and wound healing properties were investigated, confirming the enhanced healing potential of the new nanofiber compared to traditional water-based solutions [119]. Research has indicated that at elevated cell densities, scaffold pores quickly become filled, leading to growth inhibition upon cell contact and subsequent reduced proliferation [120]. The results show that nanofibers can help maintain cells and provide a suitable growth environment for fibroblasts. In the process of wound healing, the migration of fibroblasts from different sources contributes to the formation of granulation tissue and extracellular matrix, which is very important for wound healing [121].

Experiments have been conducted on the preparation of nanofiber membranes through the electrospinning of PEO 50–PDLLA50 with the incorporation of nisin. The successful electrospinning of nisin into PEO 50–PDLLA50 nanofibers has been achieved, leading to the release of nisin that exhibits inhibitory effects on Staphylococcus aureus [122]. The incorporation of growth factors into nanofibers has also been shown to expedite wound healing, with findings indicating a significant reduction in skin infection induced by Staphylococcus aureus in a mouse model [123] in a study by Cansu Ulker Turan et al. In this context, poly (ω-pentadecanolactone-δ-valerolactone)/gelatin (PDL-VL/Gel) electrospun nanofibrous membranes loaded with olive bittersweet were investigated as wound dressings for the treatment of skin infections, and based on the results of antimicrobial activity testing, 75% olive bittersweet loading was found to be suitable for the treatment of Gram-negative and Gram-positive bacterial infections in skin wounds [124]. Biodegradable multifunctional microneedles (MNs) have also been developed for the treatment of deep skin infections, in which polyvinylpyrrolidone (PVP) loaded with polymyxin B (PB) is poured to create microneedle arrays, followed by electrospraying poly(lactic-glycolic acid) (PLGA) particles loaded with curcumin (CUR) for deeper therapeutic effect [125].

### 4.3. Cutaneous Pigmentation

The cause of skin pigmentation can be attributed to the overproduction of melanin by melanocytes and the heightened transport of melanosomes from melanocytes to keratinocytes [126,127]. This heightened activity is closely correlated with excessive exposure to ultraviolet radiation and can also be influenced by endocrine and autocrine factors [128]. Melanin’s characteristic color results from the synthesis of two distinct pigments catalyzed by three enzymes: tyrosinase (TYR), tyrosinase-related protein 1 (TRP-1), and tyrosinase-related protein 2 (TRP-2) [129].

An effective approach to addressing hyperpigmentation involves targeting the initial reaction step. The competitive inhibition of TYR’s affinity for tyrosine by 4-hexylresorcinol (HR), an alternative enzyme substrate, has been proposed. HR’s catalytic activity does not generate melanin precursor molecules, thus disrupting the initial stage of melanin production [130]. Another strategy is to suppress MITF expression. Ginger oil (GO), derived from ginger rhizomes, has been identified as an inhibitor of MITF expression due to the presence of 6-shogaol and 6-gingerol [131].

**Figure 8:**
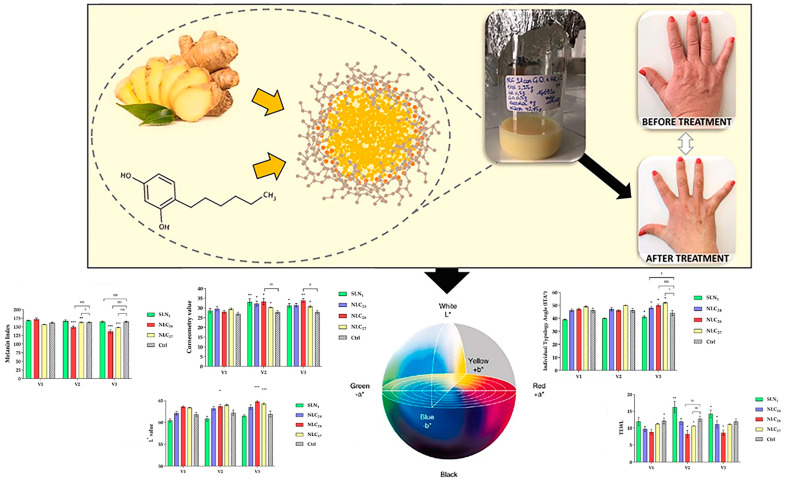
Dual delivery of ginger oil and hexylresorcinol with lipid nanoparticles for the effective treatment of cutaneous hyperpigmentation [132], with permission from Elsevier. (Difference vs. baseline *** *p* < 0.001, ** *p* < 0.01, * *p* < 0.05; difference vs. ctrl ^$$$^ *p* < 0.001, ^$$^ *p* < 0.01, ^$^ *p* < 0.05).

Combining HR and GO for dual delivery can result in additive or synergistic effects in treating skin pigmentation when incorporated into nanofiber membranes [132] (Figure 8). HR and GO have been successfully encapsulated in solid lipid nanoparticles (SLN) and nanostructured lipid carriers (NLC). GO primarily functions to inhibit tyrosinase activity and serves as both an active ingredient and an excipient in the corresponding NLC formulation. Adding GO to the lipid matrix of SLN based on Precirol can enhance the encapsulation of HR and slow down its release, leading to a more potent decolorization effect. The current evaluation demonstrates the effectiveness of SLN and NLC as delivery systems suitable for penetrating deep into the skin and gradually releasing decolorizing and whitening active components [133].

Furthermore, the nanostructured lipid carrier containing kojic acid (KA-NLC) has the ability to release KA in a controlled and sustained manner, significantly enhancing its therapeutic efficacy. This research has yielded remarkable skin improvements that could serve as a promising vehicle for managing pigmentation [134]. Similarly, Afsaneh Hoseinsalari et al. produced a novel nanostructured lipid carrier (NLC) formulation by partitioning a methanol (MeOH) extract of licorice root with ethyl acetate (EtOAc) and graded EtOAc with butanol (n-BuOH) and water, which was evaluated and concluded to have high in vivo skin penetration efficiency and anti tyrosinase activity in vivo [135]. Glycopyrrolidine (GLA) has also been developed and used to load drug combinations for the treatment of hypermelanosis via dissolving microneedles (MNs) prepared from hyaluronic acid/poly (vinyl alcohol)/poly (vinyl pyrrolidone) [136].

### 4.4. Atopic Dermatitis

Atopic dermatitis (AD), also known as eczema, is a chronic inflammatory skin condition characterized by dry and itchy skin. AD presents certain “atopy” features in patients or their family members, including a genetic predisposition to asthma, allergic rhinitis, and eczema; sensitivity to external proteins; elevated levels of IgE in the bloodstream; and an increased number of eosinophils in the blood. Typical AD showcases specific symptoms of eczema in conjunction with the aforementioned atopic characteristics. Additionally known as atopic eczema, Besnier prurigo, or hereditary allergic eczema, its treatment involves preventing bacterial infections, improving skin moisture, and reducing irritations [137].

To develop a user-friendly topical therapy, researchers utilized nanofibers as therapeutic patches due to their biocompatibility, flexibility, breathability, and healing properties. A method was created to encapsulate tacrolimus within polymer nanofiber membranes, allowing for continuous medication release. The study found that using nanofiber membranes every other day was as effective as the daily application of 0.03% tacrolimus cream, suggesting that the less frequent application of the tacrolimus-loaded nanofiber system could be a viable alternative to tacrolimus ointment [138]. Additionally, an in vivo skin hydration assessment showed that individuals with severely dry skin lacking GLA experienced increased GLA release, longer patch usage, and improved skin hydration [139].

Moreover, certain studies have demonstrated that carotenoids, particularly β-carotene, possess anti-inflammatory and healing properties alongside manganese. As a result, they were utilized in this study for eczema treatment. Hence, polycaprolactone (PCL) nanofibers were fabricated using electrostatic spinning under varying conditions, and the finest fibers were selected to encapsulate diverse concentrations of β-carotene. Fourier transform infrared spectroscopy (FTIR) confirmed the existence of β-carotene in nanofiber mats, which can be used to treat eczema, a skin disease that needs long-term treatment [140]. Pioglitazone is an oral hypoglycemic agent. In addition, it can act as a modulator of inflammatory processes and thus has been selected as a good drug for the treatment of different inflammatory skin conditions. Polyvinylpyrrolidone electrospun nanofiber patches were prepared by an electrostatic spinning technique. The application of solid microneedles enhances the total amount of drug penetration without affecting the drug penetration, resulting in high retention of the drug in the skin layer [141].

**Figure 9:**
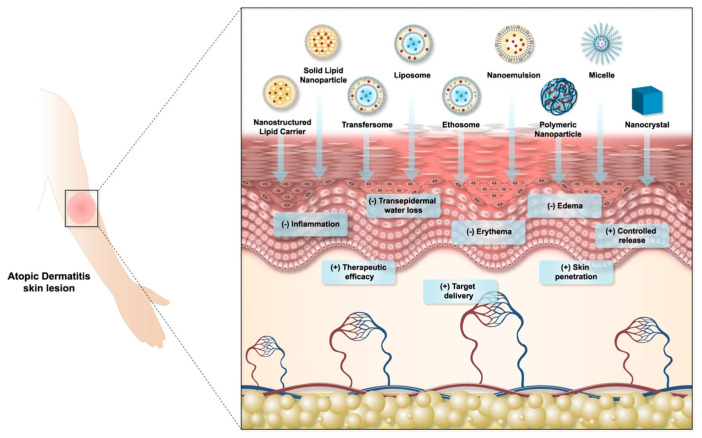
Main effects of nanocarrier-based dermopharmaceutical formulations on atopic dermatitis skin lesions [142], with permission from Elsevier.

Nanotechnology-based formulations are emerging as an alternative new paradigm for AD, aiming to improve bioavailability and delivery to the target disease site, increase drug penetration and therapeutic efficacy, and reduce systemic and off-target side effects, thus improving patient health and promoting adherence [142] (Figure 9).

### 4.5. Skin Cancer

Skin cancer, also known as tumor cells on the skin, has emerged as a significant concern attributed to the detrimental impact of ultraviolet rays penetrating the Earth’s ozone layer. The primary types of skin cancer include squamous cell carcinoma, basal cell carcinoma, and cutaneous malignant melanoma [143]. Nanotechnology presents a potential treatment approach for melanoma and skin cancer through the targeted delivery of nanofibers to the affected site, leading to decreased side effects in comparison to conventional treatments such as surgery, chemotherapy, and radiation therapy.

A team of scientists developed molybdenum nanoparticles embedded in polycaprolactone nanofiber matrices as a targeted approach to combat skin cancer. The primary objective was to induce specific tumor cell apoptosis. Using real-time polymerase chain reaction (RT-PCR) analysis of three key genes, the researchers explored the underlying mechanism. In vivo studies on zebrafish demonstrated a significant reduction in cancer progression of over 30% within a two-week period [144]. 

A nanofiber patch made of polyethylene oxide–chitosan, coated with pyrazoline-loaded carboxymethyl chitosan and dodecyl sulfate nanoparticles, was developed for the treatment of skin cancer. The fluorescence intensity of the skin increased significantly after using the nanofiber patch compared to free drugs, suggesting its potential for targeted chemotherapy against melanoma cells [145]. These results emphasize the focused therapeutic benefits of delivering anticancer drugs through nanofibers, which can help minimize the side effects often seen with systemic treatments.

Furthermore, the therapeutic potential of electrospun hybrid nanofibers in managing postoperative glioblastoma was investigated. Y zeolite nanoparticles containing curcumin were integrated into polycaprolactone–gelatin nanofiber matrices, demonstrating their potential for targeted drug delivery and enhanced treatment outcomes. Zeolite-loaded nanofibers exhibit enhanced anti-migration properties compared to drug-loaded nanofibers. Nanofiber-exposed cells demonstrate improved cytotoxicity, anti-glioma effects, and pro-apoptosis effects, suggesting the potential of incorporating curcumin-loaded Y-zeolite nanoparticles into nanofibers for glioblastoma treatment [146]. 

A synergistic approach has been developed for treating melanoma and skin cancer, utilizing coaxial nanofibers fabricated via electrospinning. The core of these nanofibers consists of chitosan-loaded polycaprolactone, while the shell comprises 5-fluorouracil (FU)-loaded poly(N-vinyl-2-pyrrolidone) [147]. This demonstrates the capability of nanofibers to synergize and offer promising strategies for skin cancer chemotherapy. Meanwhile, others have opted for topical treatment by preparing a self-heating microneedle (MN) patch with a multistage structure for rapid and sustained drug release, with a degradable tip and self-heating substrate. This self-heating MN patch demonstrated the feasibility of in vivo topical therapy for the treatment of squamous cell carcinoma of the skin [148]. It can also be used for subcutaneous delivery in a mouse model of skin tumors by NIR-responsive indocyanine green (ICG) and the chemotherapeutic agent adriamycin (Dox), which is loaded into gelatin nanoparticles (NPs) [149].

### 4.6. Psoriasis

Psoriasis, a common inflammatory skin disorder characterized by aberrant keratinocyte proliferation mediated by T cells, presents as a chronic autoimmune ailment with skin lesions exhibiting inflammatory features such as itching, pain, and bleeding. The predominant form, chronic plaque psoriasis, is triggered by genetic predisposition and various environmental factors, including stress, trauma, and infections [150,151]. 

Derived from indigo plants, indigo (IN) has been traditionally used to treat various inflammatory conditions, including psoriasis [152]. The IN-PCL/PEO nano-patch releases indigo, INdirubin, and tryptophan as active components, showcasing higher efficacy in modulating the psoriasis phenotype compared to IN ointment. This nanofiber patch alters the drug penetration and retention mechanism, leading to favorable outcomes without any adverse histological effects [153]. In addition, in the process of preparing PMVE/MA-ES nanofibers, three compounds with therapeutic activity (salicylic acid, methyl salicylate and capsaicin, with a concentration of 3.5%) were loaded into the nanofiber membrane, which can alleviate the dermatosis of psoriasis [154].

**Figure 10:**
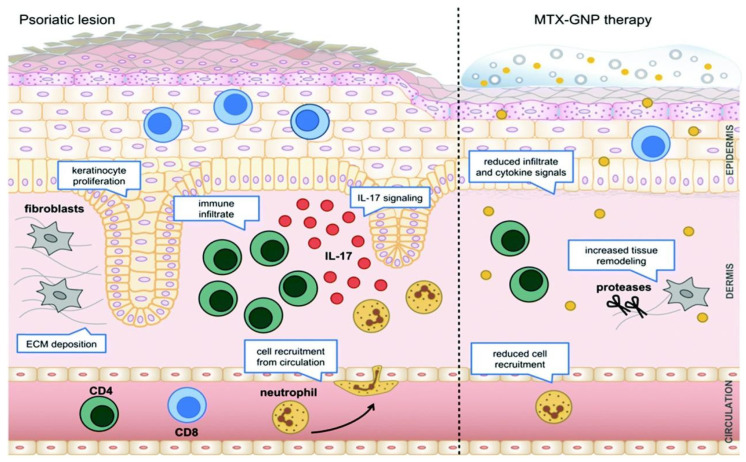
Schematic diagram of MTX coupled with AuNP to alleviate psoriasis and its potential mechanism [156], with permission from Elsevier.

In a separate trial, tazarotene was applied topically using magnetic nanoparticles enclosed within a polycaprolactone nanofiber patch. A sophisticated nanofiber setup utilizing alternating magnetic field stimulation was developed to trigger drug release, reminiscent of the gel–liquid crystal phase change observed in liposomes used for temperature-responsive drug delivery [155]. Furthermore, MTX-conjugated functionalized gold nanoparticles (AuNP) were administered in an IMQ-induced mouse model and a human xenotransplantation mouse model to assess their therapeutic impact in vivo (Figure 10). The outcomes indicated that AuNPs greatly boosted the immuno-modulatory effects of MTX, resulting in the alleviation of psoriasis lesions and reduction in the infiltration of CD45+ immune cells and IL-17 levels [156].

### 4.7. Acne

Acne, a common skin condition, is mainly caused by an imbalance in skin microflora, primarily due to the overgrowth of Dermophilus acne and Staphylococcus epidermidis, affecting individuals across different age groups. The development of acne is complex, influenced by both the composition of skin microflora and inflammatory reactions. The pathophysiology of acne includes follicular hyperkeratosis, seborrhea, and the presence of Corynebacterium acne [157].

Experiments validated the integration of essential oil into gelatin nanofibers via electrostatic spinning, creating a local antibacterial agent effective against Corynebacterium acne and Staphylococcus epidermidis, ideal for the localized treatment of acne vulgaris. The tested samples exhibited low cytotoxicity and beneficial antioxidant properties, advantageous for the targeted treatment of diverse skin infections, including common acne vulgaris [158]. To combat acne vulgaris, a novel anti-acne formulation based on chitosan nanofibers containing melittin was fabricated using electrospinning. The incorporation of melittin into the nanofibers augmented its antibacterial efficacy [159]. This nanofiber structure can be used as a suitable local drug delivery system for the treatment of acne vulgaris to provide sufficient concentration and avoid the side effects of systemic drugs.

An innovative approach was taken in the design of a warehouse formula aimed at creating a skin delivery system with enhanced permeability. This involved the use of water-insoluble polyvinyl alcohol (PVA) nanofibers incorporated with ZnO nanoparticles, which were fabricated through electrospinning technology and utilized in the production of an anti-acne mask. Nano-zinc oxide synthesized via the sol–gel method was dispersed in a solution of PVA and citric acid before being electrostatically spun to combat acne vulgaris [160]. This study demonstrates the potential of utilizing nanofibers in the development of localized drug delivery systems against acne. Moreover, a novel anti-acne formulation was devised utilizing chitosan nanofibers loaded with melittin and created through electrostatic spinning [161]. The incorporation of melittin into the nanofibers resulted in an enhanced antibacterial efficacy. The impact of Ch/Mel nanofibers on the treatment of Propionibacterium acne was evaluated using an animal model. The findings from FESEM analysis confirmed the successful achievement of the targeted nano-fiber diameter. FTIR analysis further validated the presence of Melittin within the structure. The addition of melittin to the polymer solution led to reduced conductivity, increased viscosity, a greater tensile strength in the fibers, and a decreased swelling percentage. The data showcased that the loading of 0.003% CH/MEL was 86.74%. The release kinetics of 0.003% CH/Mel (89.65%) were observed to be gradual over a period of 72 h.

**Figure 11:**
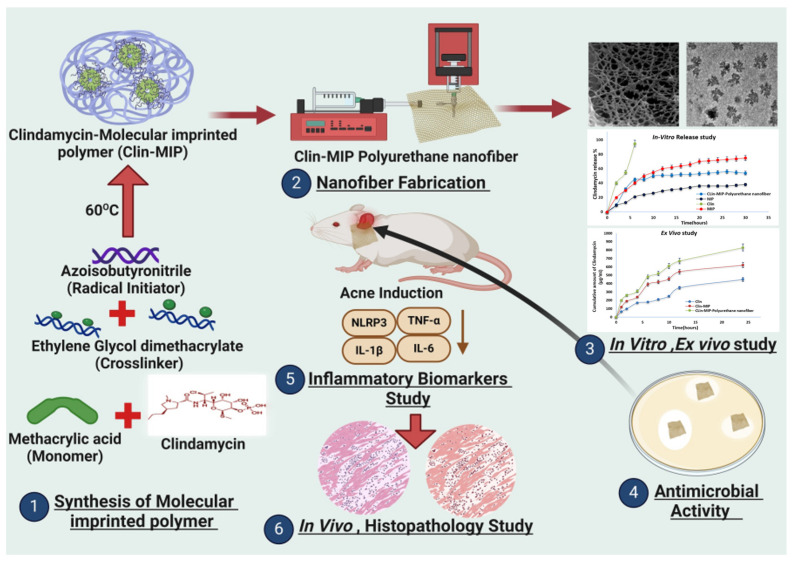
Enhanced antibacterial activity of clindamycin using molecularly imprinted polymer nanoparticles loaded with polyurethane nanofibrous scaffolds for the treatment of acne vulgaris [164], with permission from MDPI.

In addition, due to the antibacterial/antiviral activity of zinc oxide nanoparticles (NPs), which are utilized by uniformly distributing them within centrifugally spun fiber carriers, ZnO NPs can be released continuously after application to the skin and have profound antimicrobial activity at lower therapeutic concentrations to treat acne [162]. Hyaluronic acid (HA) is commonly used in cosmetic and pharmaceutical formulations, and nanofiber masks infused with AHA-BHA can be used as a topical treatment for unusual acne [163]. The topical development of a clindamycin antibiotic by molecularly imprinted technology, as well as the immobilization of clindamycin in molecularly imprinted polymers by precipitation polymerization followed by the loading of the resulting particles into polyurethane nanofibers by electrostatic spinning, can be effective in treating acne with an extended release period [164] (Figure 11).

### 4.8. Wound

Skin wounds are associated with dermal exposure, making them susceptible to infections, inflammation, and other complications that impede wound healing and could lead to severe sepsis and life-threatening conditions [165,166]. Hence, effective antibacterial and anti-inflammatory interventions are crucial in facilitating wound recovery. Various approaches have been explored to develop functional wound dressings, including nanofibers, wound patches, and hydrogels [167,168,169]. These wound dressings exhibit biocompatibility, flexibility, and the capacity to carry drugs, enabling them to prevent bacterial infections effectively and expedite the healing process.

The Janus amphiphilic fiber membrane comprises a hydrophobic layer and a hydrophilic layer. A blend of CuS NPs, PLGA, and the hydrophobic drug valsartan (V) was used for anti-inflammatory purposes, and the electrospinning method was employed to prepare the hydrophobic layer of the fiber membrane [170]. For antibacterial functions, a hydrophilic nanofiber membrane was created from PVA and the hydrophilic drug mupirocin (M). During the initial phase of wound healing, the hydrophilic layer of the Janus amphiphilic fiber membrane can steadily release hydrophilic antibacterial medications, thereby preventing infections. When granulation and growth occur, the exposure to near-infrared light can enhance the photothermal effect of CuS nanoparticles, expediting the release of anti-inflammatory drugs from the hydrophobic layer. Consequently, a adjustable gradient drug delivery system (GDR) for antibiotics and anti-inflammatory drugs can be achieved throughout the wound healing process. 

Wounds also contain a multitude of drug-loaded components. Naringin was incorporated into polycaprolactone (PCL)/polyethylene glycol (PEG) nanofibers (NFs) to create a novel antibacterial wound dressing [171]. An MTT assay demonstrated an increase in the viability of human fibroblasts, confirming the non-toxicity of the material. In a rat wound injury model, the Nrg-loaded dressing exhibited re-epithelialization and wound closure similar to that of commercially available phenytoin ointment. Furthermore, a dual-layer skin scaffold loaded with drugs was developed for mending full-thickness skin defects [172]. Amoxicillin (AMX) was loaded onto polycaprolactone (PCL) nanofibers using electrostatic spinning, forming an antibacterial nanofiber membrane (PCL-AMX) as the outer layer of the scaffold to mimic the epidermis. In order to maintain wound wettability and promote wound healing, external human epidermal growth factor (rhEGF) was loaded into sodium alginate–gelatin, and a hydrogel structure (SG-rhEGF) was formed by 3D printing, which was used as the inner layer of scaffold to simulate dermis, which could enhance cell adhesion and proliferation and promote skin wound healing, and had good biocompatibility.

**Figure 12:**
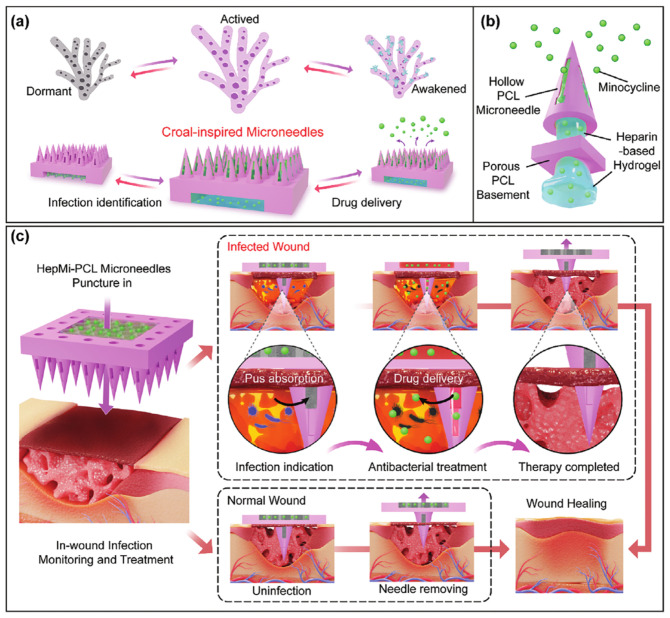
Hollow microneedle patch for chronic infected wounds [173], with permission from Wiley. (**a**) The design inspiration for porous and hollow microneedles comes from the structure and biological behavior of corals. (**b**) The inside room of the porous microneedle shell is filled with drug carrying hydrogel. The bottom opening window of microneedle is placed on the back of the patch. (**c**) The microneedle patch could penetrate the scab shell on the surface of the wound and collect exudate from the tissue.

In addition, a bionic microneedle patch (HepMi-PCL) was developed to indicate and treat infection within the wound. Relying on high-precision 3D printing technology, each polycaprolactone microneedle has an equal-sized porous shell and cavity filled with a heparin-based functional hydrogel. After penetrating the scab, HepMi-PCL absorbs wound exudate on its microneedles through surface guide grooves and pore microstructures, thus indicating the need for anti-infection. Once a positive chronic infection is detected, HepMi-PCL is intelligently activated for rapid drug release [173] (Figure 12).

### 4.9. Other Diseases

Scarring occurs as a result of damage to the skin and soft tissues caused by physical, biological, and chemical factors. This damage cannot be fully self-repaired and is replaced by fibrous tissue, affecting both appearance and functionality. The use of dressings is essential in the healing of acute and chronic wounds [174].

Optimal wound dressings need to maintain a moist healing environment, allow for gas exchange, absorb wound secretions, prevent skin maceration, and offer waterproof protection against harm or infection [175]. Electrospun nanofiber dressings made from biopolymer materials are a cost-effective, biocompatible, and straightforward option for wound care [176]. Research has shown that these dressings promote cell proliferation and migration within the wound bed, as well as supporting the hemostasis, gas exchange, and management of wound secretions.

Utilizing electrospinning technology, four distinct medical-grade polyester, polycarbonate, and polyurethane polymer formulations were developed to create customized nanofiber dressings tailored to specific wound characteristics and stages of healing. These personalized dressings have alleviated pain, minimized scarring, reduced the need for dressing changes, and enhanced cost efficiency. Electrospun polymer nanofiber dressings offer a noninvasive, viable, and secure approach for managing partial-thickness wounds. They can potentially expedite wound recovery and decrease the risk of infection [177]. Furthermore, experimental evidence supports the effectiveness of nanofibers in scar treatment. Encapsulating dextran with clove oil (CO) and sandalwood can inhibit the antibacterial activity of infected oil (SO), thus enhancing the wound healing process and accelerating scar-free recovery. The inherent benefits of nanofibers suggest promising outcomes in improving wound healing and reducing scar formation. A dextran/nano-soy/glycerol/chitosan (DNG/Ch) nano-composite membrane was prepared by simple solvent casting technology, and then CO and SO were added to obtain a herbal nano-dressing [178].

## 5. Conclusions and Prospects

Significant progress has been made in the use of electrospun nanofibers for external pharmaceutical applications. Electrospinning is an efficient technique for producing nano-sized fibers and high-porosity nanofiber membranes with pores ranging from nano to micro scales. Various medications, including anti-cancer drugs, proteins, antibiotics, RNA, and DNA, have been incorporated onto electrospun nanofibers [179]. Methods such as embedding drugs within electrospun nanofibers and applying drugs onto their surfaces have been used to create electrospun nanofiber scaffolds, acting as nano-carriers for medications. These approaches help achieve controlled and sustained drug delivery at specific sites by adjusting drug release kinetics [179]. In addition to enabling controlled drug release, electrospinning technology also improves therapeutic outcomes and reduces toxicity levels. A wide range of drugs can be incorporated into electrospun nanofibers. Researchers worldwide have extensively studied the effectiveness of electrospun nanofibers as drug delivery systems, evaluating the effects of degradable and non-degradable polymers on sustained drug release through electrospun nanofibers [180,181].

Nano-fiber films have found widespread applications, particularly in evaluating in vivo stability, durability, and biocompatibility across various fields. These attributes are crucial in medical settings for tasks like biosensors, wound healing, tissue regeneration, and drug delivery. Challenges also arise in utilizing electrospun nanofibers for energy devices due to difficulties in the large-scale production of nanofibers with the desired properties. Issues stem from the limitations of traditional electrospinning methods, including low efficiency, high voltage requirements, and complexities in on-site nanofiber deposition [182]. Additionally, cell penetration in electrospun nanofiber scaffolds remains a concern, but advancements are being made in creating scaffolds with improved cell infiltration for use in 3D structures. Enhancing the structure of nanofibers demands significant energy and expertise to enable the better integration of nanofiber films in treating skin conditions, leveraging their wound healing capabilities. This progress has paved the way for exploring diverse polymer matrices and drugs for wound healing applications. Consequently, drug-loaded nanofiber membranes present a novel opportunity and challenge in treating external wounds like skin diseases, offering substantial value for potential commercialization on a larger scale.

## Figures and Tables

**Table 1:** Application of electrostatic spinning technology in skin diseases.

Types of Electrostatic Spinning Technology	Carbohydrate Types	Superiority	Treatment of Dermatosis Category	References
Coaxial electrostatic spinning	Specific protein polymer	Has higher cell proliferation efficiency; can change the surface characteristics; multi-system solution spinning; can easily manufacture nanofibers; easy to operate; excellent material handling ability;	Skin burning	[34]
Side-by-side electrostatic spinning	Synthetic polymer	The two solutions can be blended, and the release effect in vitro is remarkable; productivity can be improved.	Diabetic wound	[35]
Triaxial electrostatic spinning	Synthetic polymer	Able to build complex drug control system; promote the dissolution and penetration of drugs with poor water solubility in the model.	Diabetic ulcer	[36]
Emulsion electrostatic spinning	polysaccharide	Easy to process and control; suitable for delivering hydrophobic and hydrophilic drugs.	Skin wound	[37]
Multi-nozzle electrostatic spinning	polysaccharide	Manufactures large nanofibers to increase yield and coverage.	Skin wound	[38]
Portable electrostatic spinning	Synthetic polymer	Flexible use; in situ spinning; higher voltage; accuracy of voltage and flow; safe use; precise deposition	Skin burning	[39]
Near-field electrostatic spinning	Synthetic polymer	Combining biological 3D printing with traditional disordered electrospinning technology, highly ordered ultrafine fibers can be prepared.	Diabetic wound	[40]

**Table 2:** Classification and application of nanofiber membranes.

Classify	Type	Medicine Carrying	Main Findings	References
Coaxial electrostatic spinning	PVP/PLA nanofibers	Astragaloside iv	Promote the healing of diabetic wounds	[86]
	PEO-CS-LEV/PLGA-QS core–shell nanofibers	Levofloxacin	Used for burn wound healing	[87]
	PHB+SAL-PEO core–shell nanofiber mat	Embelin	Wound healing	[88]
	PCL shell and DOPA coating	Double growth factor (EGF/bFGF)	Wound healing	[89]
	PVP core layer combined with CS-PCL as shell layer.	rheum emodin	Skin cancer	[90]
Side-by-side electrostatic spinning	Core–shell nanofibers of F127-Mup/Pec-Kr	Mupirocin	Wound dressing	[91]
	Multifunctional medicinal three-segment Janus nanofiber	Beeswax, Quercetin and Ketoprofen	Anti-adhesion repair of tendon	[92]
	Janus nanofibers	Zinc oxide nanoparticles and curcumin	Wound dressing	[93]
Triaxial electrostatic spinning	Beaded (BOTS) microfibers	CURcumin	Improve colon-targeted drug delivery	[94]
	Cellulose acetate	Ferulic acid	Drug delivery	[95]
	Core–shell nanofibers (CSF) of Eudragit S100 (ES100)	Aspirin	Colon targeting prolongs drug release	[56]
	Protein nanocomposites-a drug coated with cellulose acetate	Ibuprofen	Regulating drug release	[96]
Multi-nozzle electrostatic spinning	Poly ε-caprolactone (PCL), zein and gum Arabic (GA)	*C. officinalis*	Skin tissue engineering	[97]
	PCL/gelatin nanofibers	CURcumin	Skin application	[98]
	Polycaprolactone/chitosan-polyethylene oxide (PCL/Cs-PEO)	*A. euchroma*	Application of skin tissue engineering	[38]
Needle-free electrostatic spinning	Collagen/hyaluronic acid nanofibers	Collagen	Skin moisturizing	[99]
	Polycaprolactone (PCL) and poly (vinyl alcohol) (PVA) nanofibers (PCL-PVA)	Platelet lysate	Chronic wound	[100]
Emulsion electrostatic spinning	Poly (L-lactic acid) (PLLA)/poly (vinyl alcohol) (PVA)/chitosan (CS)	*Hypericum perforatum*	Wound dressing	[101]
	PLA nanofiber membrane	*Bletilla tuber*	Diabetic wound	[102]
	bFGF-ATP-Zn/PCL nano-dressing	Basic fibroblast growth factor (bFGF)	Relieve wounds and scars	[103]
	PVA/RES/PR nano-materials	Resveratrol	Skin moisturizing mask	[104]

## Data Availability

Data supporting the findings are available upon reasonable request to the corresponding authors.
